# Confocal Laser Endomicroscopy for *In Vivo* Diagnosis of *Clostridium difficile* Associated Colitis — A Pilot Study

**DOI:** 10.1371/journal.pone.0058753

**Published:** 2013-03-19

**Authors:** Helmut Neumann, Claudia Günther, Michael Vieth, Martin Grauer, Nadine Wittkopf, Jonas Mudter, Christoph Becker, Christoph Schoerner, Raja Atreya, Markus F. Neurath

**Affiliations:** 1 Department of Medicine I, University of Erlangen-Nuremberg, Erlangen, Germany; 2 Institute of Pathology, Klinikum Bayreuth, Bayreuth, Germany; 3 Institute of Microbiology, Immunology and Hygiene, University of Erlangen-Nuremberg, Erlangen, Germany; Charité, Campus Benjamin Franklin, Germany

## Abstract

**Background:**

*Clostridium difficile* infection (CDI) is one of the most dreaded causes of hospital-acquired diarrhea. Main objective was to investigate whether confocal laser endomicroscopy (CLE) has the capability for *in vivo* diagnosis of *C. difficile* associated histological changes. Second objective was to prove the presence of intramucosal bacteria using CLE.

**Methods:**

80 patients were prospectively included, 10 patients were diagnosed with CDI based on toxigenic culture. To validate the presence of intramucosal bacteria *ex vivo*, CLE was performed in pure *C. difficile* culture; additionally fluorescence *in situ* hybridization (FISH) was performed. Finally, CLE with fluorescence labelled oligonucleotide probe specific for *C. difficile* was performed *ex vivo* in order to prove the presence of bacteria.

**Results:**

CLE identified CDI-associated histological changes *in vivo* (sensitivity and accuracy of 88.9% and 96.3%). In addition, intramucosal bacteria were visualized. The presence of these bacteria could be proven by CLE with labeled, specific molecular *C. difficile* probe and FISH-technique. Based on comparison between CLE and FISH analyses, sensitivity and specificity for the presence of intramucosal bacteria were 100%.

**Conclusion:**

CLE has the potential for *in vivo* diagnosis of CDI associated colitis. In addition, CLE allowed the detection of intramucosal bacteria *in vivo*.

## Introduction

*Clostridium difficile* infection has emerged as one of the most clinically significant causes of hospital-acquired diarrhea and is associated with significant morbidity and mortality. *C. difficile* infection is often accompanied by fever and leukocytosis and frequently affects older and immunocompromised patients. Nevertheless, recent data suggest that even young and healthy persons who have previously not been exposed to a health care environment or antimicrobial therapy are at risk as well [1], [2].

*C. difficile* can colonize the large bowel and, in the presence of antibiotic therapy that limits the growth of naturally residing microorganisms, produce endotoxins and cytotoxins that can cause severe mucosal damage, resulting in colitis that may have a pseudomembranous appearance at endoscopy [1]. It has been estimated that *C. difficile* causes approximately 25% of the antibiotic-associated diarrhea (AAD) and most cases of pseudomembranous colitis. In the United States, there are about 300,000 cases of *C. difficile*-associated diarrhea and colitis per year, resulting in an annual economic burden of more than one billion dollars to the health care system [2].

Since a new epidemic strain of *C. difficile* associated with more severe disease, mortality and frequent relapses has been identified in 2003, *C. difficile* infection has becoming increasingly difficult to control and eradicate [3]. Accordingly, *C. difficile* infection now rivals methicillin-resistant *Staphylococcus aureus* (MRSA) as the most common cause for hospital-acquired infections in the United States.

Therefore, rapid and accurate diagnosis of *C. difficile* infection is of crucial importance, not only for individual patient management but also for prevention of nosocomial transmission. Currently, diagnosis of *C. difficile* infection is based on patients' clinical history and laboratory tests, including toxigenic culture, which still remains the gold standard for diagnosis [4].

Recently, confocal laser endomicroscopy has been introduced as a new endoscopic imaging technique enabling real time *in vivo* histology of the cellular and subcellular mucosal layer at a magnification of 1000 fold. Previously it was shown that endomicroscopy has the capability to facilitate histopathological diagnosis of different gastrointestinal diseases including Barrett's esophagus, celiac disease, microscopic colitis and inflammatory bowel diseases [5]–[11].

Our main study objective was to prospectively investigate whether CLE has the capability for the *in vivo* diagnosis of *C. difficile* associated histological changes. Second objective of our study was to prove the presence of *C. difficile* bacteria using CLE.

## Patients and Methods

### Patients

Consecutive patients, including both, in-patients and out-patients with diarrhea who underwent colonoscopy for the evaluation of their symptoms were prospectively included between October 2009 and September 2010. All patients signed informt consent to participate in this study after the endoscopist and attending physician had explained the procedure in detail to them. Subjects were enrolled if they met the following inclusion criteria: more than 18 years of age, ability to provide written informt consent, diarrhea. Patients with one or more of the following criteria were excluded from the study: inability to provide written informt consent, severe uncontrolled coagulopathy, impaired renal function, pregnancy or breast feeding, active gastrointestinal bleeding, known allergy to fluorescein or acriflavine and residing in institutions (e.g. prison).

The study was approved by the local ethical committee and government authorities (IRB approval of the University of Erlangen-Nuremberg; http://www.ethik.med.uni-erlangen.de/) and was conducted according to the declaration of Helsinki. The UMIN Clinical Trials Registry identification number for this study was NCT01072110. Written informt consent was obtained from all participants. Clinical data, including patients' history and laboratory data were recorded. Final diagnosis of *C. difficile* infection was based on toxigenic culture. Two endoscopists and one experienced gastrointestinal pathologist read the images. Endoscopists and pathologist were blinded to all microbiological results.

### *In vivo* confocal laser endomicroscopy in *C. difficile* patients

Patients underwent a standard bowel preparation using either oral sodium phosphate or polyethylene glycol-electrolyte lavage solution. All endomicroscopy procedures were performed under conscious sedation with constant monitoring of vital signs using either integrated-endomicroscopy (iCLE; EC-3870CILK, Pentax, Tokyo, Japan) or probe-based endomicroscopy (pCLE; Cellvizio, ColoFlex^UHD^, Mauna Kea Technologies, Paris, France). Both systems use an incident 488 nm wavelength laser (blue laser light) and enable the detection of fluorescence between 205 – 585 nm wavelengths [9]. Randomization was performed as followed: Patients were randomized into both groups by using a computer-aided system. The results of the randomization were kept in sealed envelopes that were opened just before the endoscopic procedure. Authors were blinded to diagnosis of *C. difficile* infection. In every patient, 10 localisations were analysed. In case of mucosal abnormalities, like erythema or ulcers, CLE was performed at these places. In case of macroscopically normal appearing mucosa, CLE was performed in the rectum (proximal and distal part), sigmoid colon (proximal and distal part), descending colon (proximal and distal part), transverse colon (proximal and distal part), ascending colon und caecum. During CLE *in vivo* diagnosis of CDI was either made or excluded. Therefore, 100 lesions were examined in CDI patients and 700 in control patients which were not affected with CDI. iCLE images were collected at a frame rate of 0.8/s at 1024×1024 pixels or 1.6/s at 1024×512 pixels. Intravenous fluorescein was used in every patient to optimize tissue contrast. Additionally, in some patients topical acriflavine hydrochloride (0.05%; Sigma-Aldrich, Steinheim, Germany) or topical cresyl violet (0.13%; Alcon Laboratories, Texas, USA) was applied after intravenous injection of fluorescein using standard spraying catheters (Olympus, Tokyo, Japan). First, confocal images were analyzed in real time. Subsequently, images were reviewed offline to digitally zoom in on details (iCLE), allowing a higher magnification of the mucosa (approximately 10,000 fold) or by using the Cellvizio Viewer (Mauna Kea Technologies, Paris, France) for virtual staining of mucosal structures to enhance tissue contrast.

Normal mucosa and pathological lesions were evaluated according to the Mainz confocal pattern classification for iCLE and according to the modified Miami classification for pCLE [12], [13].

After endomicroscopy, biopsies were taken from macroscopically normal and altered mucosa and specimens were fixed in 4% buffered formalin for subsequent histopathological analysis. In case of macroscopically normal mucosa random biopsies were performed from all evaluated colon segments (e.g. terminal ileum, caecum, ascending colon, transverse colon, descending colon, sigmoid colon, and rectum). In case of macroscopically visible lesions targeted biopsies were performed from these areas.

In order to reach total concordance between *in vivo* iCLE-imaging and biopsy acquisition, mild suctioning was applied to the mucosa for confocal imaging. Technically, the resulting mucosal hemorrhage was located 5 mm to the right from the area which had been evaluated using iCLE. After performing pCLE, the probe was gently pushed against the mucosal wall, thereby marking the area. Thus, the exact correlation between *in vivo* imaging and biopsy acquisition was determined.

### Ex-vivo endomicroscopy of C. difficile

First, the *C. difficile* strain was cultured on *C. difficile* selective agar and harvested. Subsequently, the pure *C. difficile* culture was resuspended in physiological NaCl. Afterwards, a diluted solution of 0.05% acriflavine hydrochloride or fluorescein sodium was added and the solution was subsequently imaged using the integrated confocal laser endomicroscopy system.

In addition, biopsy samples were obtained from two patients with *C. difficile*-infection and subsequently incubated with phosphate buffered saline (PBS). Afterwards, a 5% solution of fluorescein-labelled *C. difficile* specific probe was added and the biopsy specimens were subsequently imaged using the integrated confocal laser endomicroscopy system (Five 1, Optiscan, Notting Hill, Australia).

Endomicroscopy images were collected at a frame rate of 0.8/s at 1024×1024 pixels and digitally magnified after image acquisition at 10,000 fold to further zoom in on details.

### Fluorescence *in Situ* Hybridization (FISH) of *C. difficile*

FISH was performed on biopsies from different areas of the sigmoid colon of four patients, two control patients and two patients infected with *C. difficile*. FISH staining of bacterial rRNA on glass slides was performed as previously described [14]–[16]. In short, FISH hybridization of bacterial rRNA was performed on 3 µm cross sections of formalin fixed paraffin embedded biopsies on glass slides. Slides were dewaxed and rehydrated by incubating them for 30 minutes at 60°C and additional 3 cycles of xylol (each 5 minutes), 2 cycles of 100% ethanol (each 3 minutes), 1 cycle of 96% ethanol (3 minutes), 1 cycle of 70% ethanol and 1 cycle of distilled water (3 minutes). Afterwards, paraffin cross sections on glass slides were shortly washed with PBS and preincubated using hybridization buffer containing 20% formamide for 10 minutes. For the detection of bacterial rRNA, samples were incubated with 47.5 ng of a Cy3-labeled *C. difficile* specific probe in 50 µl of hybridization buffer containing 20% formamide for 90 minutes at 46 °C. Finally, the paraffin cross sections were washed with incubation buffer for 15 minutes at 46°C and afterwards shortly with PBS. Nuclear counterstaining was performed using Hoechst dye 3342. The *C. difficile* specific probe (Cd-198 m; 5′ CAT CCT GTA CTG GCT CAC) was previously designed in a study by Bloedt and coworkers [17]. We also refer to this study regarding the evaluation of the FISH probe for *C. difficile* and the phylogenetical analysis.

### Statistical Analysis

The statistical software program PASW Statistics 18 (SPSS, Inc., Chicago, USA) was used for all data analysis. Final statistical analysis for *in vivo* histology was based on the results of *in vivo* CLE and the results of the toxigenic culture based on a per-patient analysis. The *t*-test was used for all continues variables to determine whether differences between any two groups existed. A two-sided P value <0.05 was considered to be significant. To evaluate the impact of image interpretation for diagnosis of CDI we calculated the positive and negative predictive values. In addition, the sensitivity, specificity and accuracy of the endomicroscopy findings were also calculated. The median in this study is presented for non-normally distributed variables, and the mean for normally distributed variables. The range indicated the range between the minimum and maximum values. Correlation between endomicroscopy diagnosis and histopathology was determined using kappa statistics, which assesses agreement beyond chance among investigators. Therefore, the strength of rater agreement was categorized according to the definition proposed by Landis and Koch for kappa values [18]: 0 – 0.20, slight; 0.21 – 0.40, fair; 0.41 – 0.60, moderate; 0.61 – 0.80, substantial; 0.81 – 1.00, almost perfect.

## Results

During the study period, a total of eighty patients with diarrhea were prospectively included. In ten (4 female, 6 male; mean age 72.5 years, range 37 – 96 years) out of these eighty patients *C. difficile* infection (CDI) was diagnosed as the constitutive cause for diarrhea based on toxigenic culture as the gold standard. Remaining causes for diarrhea included infectious pathogens other than CDI and inflammatory bowel diseases.

### High-resolution endoscopy

**Figure 1:**
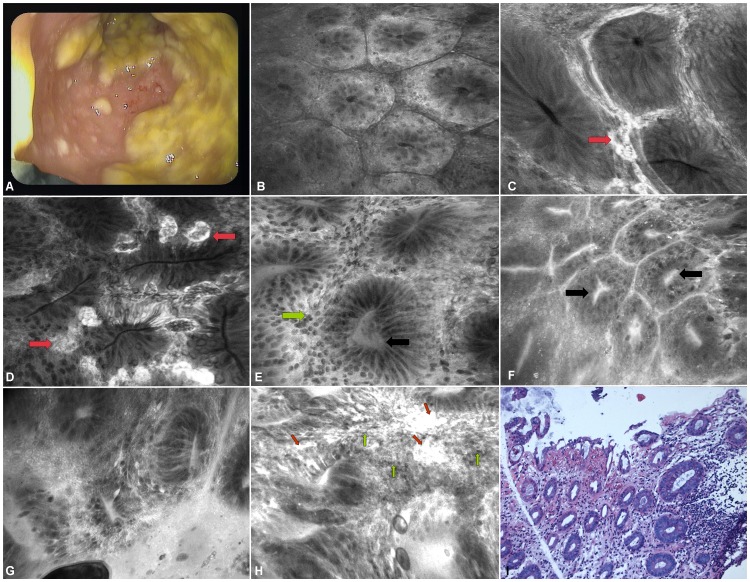
Endoscopic and endomicroscopic appearance of C. difficile infection. Panel A highlights the macroscopic appearance of advanced *C. difficile* infection using high-resolution endoscopy. Confluent cream to yellow coloured plaques varying in size, which were only loosely attached to the colon wall, were seen. The underlying mucosa was hyperaemic. Note the confocal lens of the integrated-endomicroscopy system at the 7 o'clock position. Panel B shows normal colonic architecture in disease-free mucosa as control (×1000). Dependent on the extent of disease manifestation, colonic crypts in *C. difficile* colitis were variously shaped (black arrows) (Panels C-F; ×1000). Furthermore, microvessels (red arrows) were dilated but showed no signs of leakage in milder forms of colitis (Panel C-F; ×1000). Furthermore, an increased cellular infiltrate (green arrows) within the lamina propria became visible in *C. difficile* colitis (Panel E; ×1000). Fluorescein-aided endomicroscopy of advanced *C. difficile*-infection (Panel G, H; ×1000) showed fragile vessels with fluorescein leakage (red arrows) and dense cellular infiltrates (green arrows) in the lamina propria. In these cases, the normal colonic architecture was nearly completely abolished. Histopathological assessment of mild *C. difficile* infection is shown in panel I (×200).

On high-resolution white-light endoscopy (EC 3840FK2, Pentax, Tokyo, Japan) the colonic mucosa was covered with confluent pseudomembranes and showed linear ulcers in two out of ten patients with CDI. In three patients, discrete cream to yellowish coloured plaques, varying in size between 2 to 30 mm were visualized [Figure 1]. The plaques were only loosely attached to the colon wall and the underlying mucosa was hyperaemic. In five patients, only a slightly erythematous mucosa without any plaques was visible.

### *In vivo* confocal laser endomicroscopy

Overall, 100 lesions were examined using CLE in CDI patients and the results were compared to 700 locations of control patients as mentioned in the “Patients and Methods” section. According to disease manifestation, different characteristics of *C. difficile* infection were found. Following intravenous injection of fluorescein, at early disease stages without macroscopically visible pseudomembranes, small surface erosions of the superficial colonic crypts were visualized. Additionally, an increased cellular infiltrate within the lamina propria was visible. Microvessels within the lamina propria were slightly dilated but showed no leakage [Figure 1]. The average distance between colonic crypts was weakly augmented, indicating mucosal edema. On the luminal side, endomicroscopy demonstrated mucus, fibrin and epithelial cells. These areas were surrounded by normal appearing mucosa.

In advanced disease stages of CDI with macroscopically visible pseudomembranes, confocal laser endomicroscopy demonstrated massively dense cellular infiltrates within the lamina propria. Colonic crypts were variously shaped and irregular in arrangement. Normal colonic appearance was nearly completely abolished. According to the degree of inflammation, fragile vessels and leakage demonstrated by extravasation of fluorescein became visible [Figure 1]. Furthermore, a plaque of loosely cells, fibrin and debris covered the mucosal surface.

Sensitivity, specificity, and accuracy of endomicroscopy to determine CDI-associated histological changes *in vivo* were 88.9%, 97.2%, and 96.25%, respectively. Positive and negative predictive values were calculated as being 80.0% and 98.6%, respectively. Correlation between endomicroscopy and histopathology was good (kappa 0.8209; 95% confidence interval 0.6221 – 1.00).

**Figure 2:**
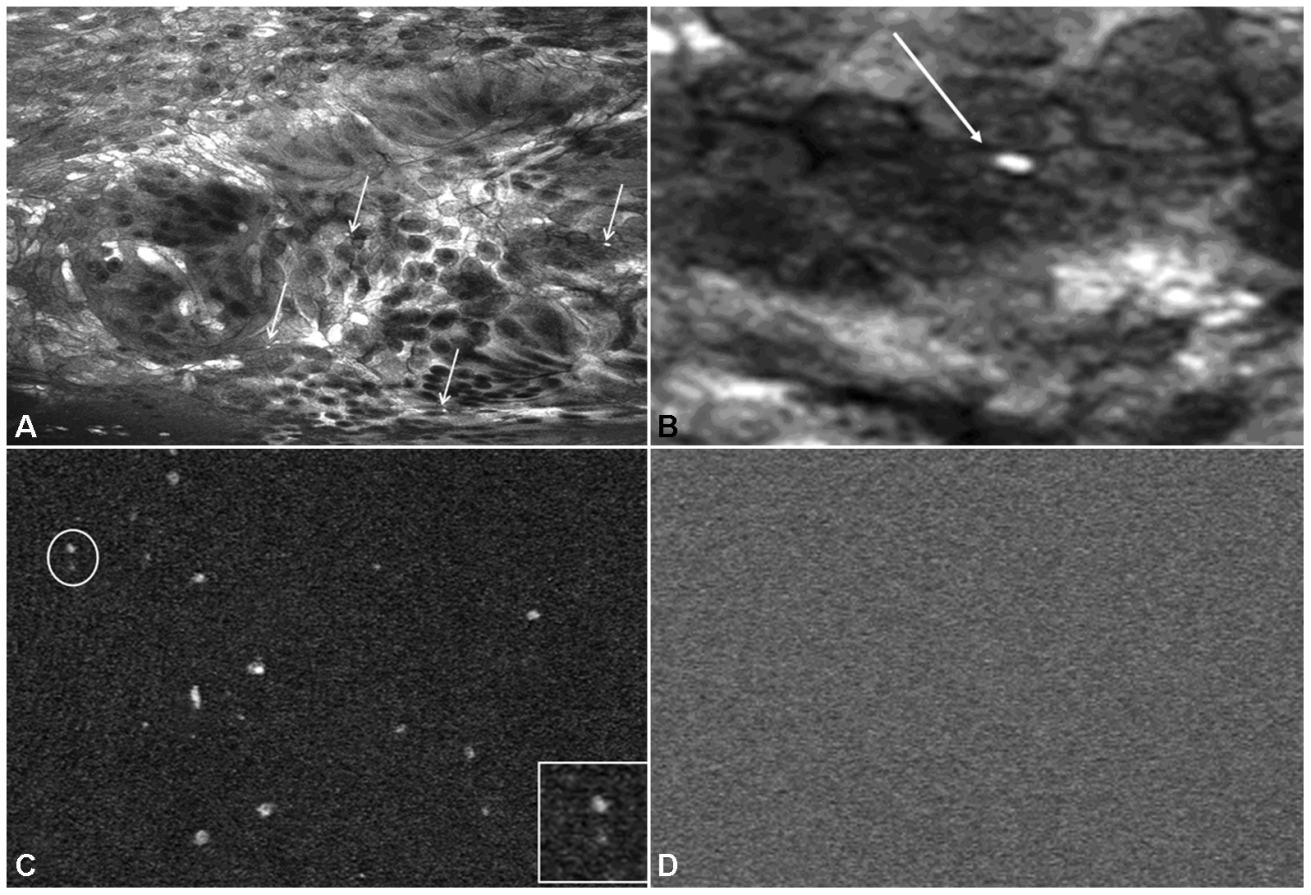
*In vivo* visualization of intramucosal bacteria within the colonic mucosa in *C. difficile* colitis by confocal laser endomicroscopy. Fluorescence confocal image below the surface of the colonic mucosa after topical application of acriflavine hydrochloride identified single bacteria (Panel A, arrows). At 10,000 fold digital magnification the rod-like appearance of bacteria (arrow) in the colonic mucosa became visible (Panel B). Panel C shows e*x vivo* imaging of pure cultured *C. difficile* at 1000-fold magnification and 10,000 fold magnification (insert in lower right quadrant) after staining with acriflavine hydrochloride. In contrast, after application of fluorescein no bacteria were visible by confocal imaging (Panel D).

Fluorescein-based CLE has recently been described as a new technique to identify translocating bacteria in the mucosa of patients with inflammatory bowel diseases suggesting that this approach has the capacity to identify mucosal bacteria [19]. Surprisingly, in this study no specific bacteria translocating into the mucosa could be identified in CDI using fluorescein-aided CLE. However, in contrast to fluorescein-aided CLE, white focal spots in the colonic mucosa and the pericryptal space could be identified after topical application of acriflavine hydrochloride [Figure 2]. Image review of confocal images at 10,000 fold digital magnification revealed a rod like appearance of the white focal spots suggesting the presence of *C. difficile* bacteria [Figure 2]. In contrast to acriflavine, the topical application of cresyl violet did not add any additional information compared to single fluorescein staining. No adverse events regarding the procedure or the use of the different dye agents were observed.

### *Ex vivo* confocal laser endomicroscopy

Consistent with the above *in vivo* findings, fluorescein-aided *ex vivo* endomicroscopy of pure *C. difficile* culture revealed no noticeable structures [Figure 2] suggesting that these bacteria lack the potential for fluorescein uptake. In contrast, e*x vivo* endomicroscopy of pure *C. difficile* culture after application of acriflavine hydrochloride revealed white focal spots comparable to the *in vivo* confocal imaging. In addition, image review at 10,000 fold digital magnification confirmed the rod like appearance of the white focal lesions in the presence of *C. difficile* [Figure 2].

**Figure 3:**
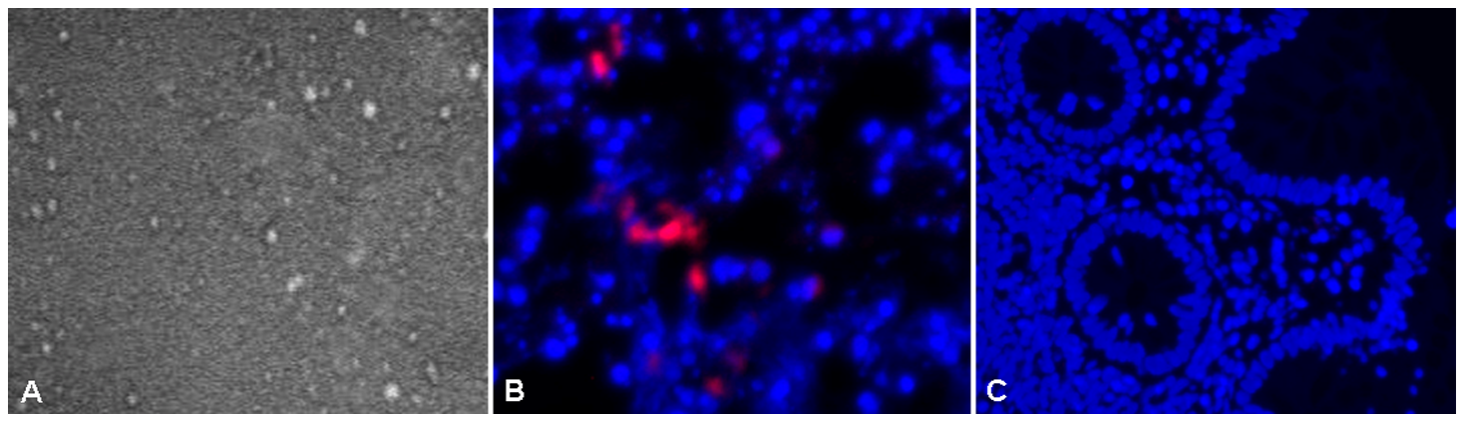
*Ex vivo* verification of *C. difficile* using a fluorescence labelled *C. difficile* specific probe in CDI. Panel A shows a characteristic endomicroscopic image of a patient with *C. difficile*-infection after exposure of the biopsy to the labelled probe and subsequent CLE analysis. Intramucosal bacteria were clearly visible (white spots). Fluorescence in situ hybridization (FISH) confirmed the presence of intramucosal bacteria in this patient due to the bright red fluorescence. Nuclei and DNA are displayed in blue (Panel B). Panel C shows FISH in a control patient without *C. difficile* infection. No bacteria could be visualized.

To prove the potential of CLE for the identification of *C. difficile* bacteria, we performed a final series of studies with *ex vivo* CLE of colonic biopsies with a labeled specific probe for *C. difficile* [17]. Interestingly, *ex vivo* CLE with a fluorescein-labelled *C. difficile* specific probe visualized intramucosal bacteria within the colonic epithelium similar to *in vivo* endomicroscopy and FISH-technique [Figure 3] indicating that CLE with molecular probes could potentially be used to specifically identify *C. difficile* bacteria.

### Fluorescence in Situ Hybridization (FISH) of intramucosal bacteria

To provide additional validation for the identification of intramucosal *C. difficile* bacteria at endomicroscopy, we performed FISH of intestinal biopsies which were taken from areas previously evaluated using endomicroscopy. In all biopsies from CDI patients, FISH confirmed the presence of intramucosal bacteria in the colonic mucosa and the pericryptal space [Figure 3]. These bacteria corresponded to the focal spots which were previously imaged by *in vivo* and *ex vivo* endomicroscopy. Sensitivity and specificity for the presence of intramucosal bacteria detected by CLE were 100% when compared to FISH findings.

## Discussion

In this pilot study, we have identified confocal laser endomicroscopy (CLE) as a potential novel technique for the diagnosis of *C. difficile* infection (CDI). CLE enabled real time, *in vivo* diagnosis of CDI associated histological changes regardless from the disease stage. Additionally, acriflavine-aided endomicroscopy was able to visualize intramucosal *C. difficile* bacteria within the lamina propria. The presence of intramucosal *C. difficile* bacteria was additionally proven using *ex vivo* CLE with specific molecular probe and FISH technique. The finding of intramucosal bacteria in our study did not correlate to the clinical symptoms of the patients.

Endomicroscopy is a new, emerging endoscopic imaging modality enabling real time *in vivo* histology during ongoing endoscopy. Currently, two FDA approved devices for endomicroscopy are available [9]. While one device is integrated into the distal tip of a high-resolution endoscope (iCLE, Pentax, Tokyo, Japan), the other device represents a stand-alone confocal miniprobe which is capable of passage through the accessory channel of a standard endoscope (pCLE, Cellvizio, Mauna Kea Technologies, Paris, France). In our study, we used both systems for confocal diagnosis. Although it was not the purpose of this study to compare both techniques, both confocal imaging systems could readily identify architectural changes in *C. difficile* infection without obvious differences. Nevertheless, for the first time both endomicroscopy systems were evaluated in one study.

*C. difficile* associated colitis is known to lead to a superficial inflammation and is therefore ideally suited for CLE based analysis [20]. In fact, we were able to observe similar changes by CLE as compared to standard histopathology with high sensitivity, specificity and accuracy. In fact, the correlation between real time *in vivo* histology and *ex vivo* histology in predicting CDI-associated histological changes was good. In comparison, assessment of toxins A and B by ELISA has a sensitivity of 63–99% and a specificity of 93–100% with a PPV of 73% and a NPV of 96% [21]. As CLE allows on demand *in vivo* diagnosis of cellular and subcellular structures in real time during ongoing endoscopy CLE may have the potential to provide a faster diagnosis of CDI as compared to conventional culture. However, our approach needs further validation by a prospective multicenter study with a respective sample size calculation.

Our study has potential limitations. First, although the investigators had long experience in performing and interpreting endomicroscopy, this may not have been sufficient to reliably obtain and interpret endomicroscopy images in CDI. Second, endomicroscopy images do not represent typical histological images, as they illustrate a horizontal field of view. Therefore, it is possible that additional new criteria for CDI-associated colitis on horizontal sections have to be defined. Third, infectious colitis caused by other pathogens may also mimic CDI-associated endoscopic and histologic changes [22]. Finally, although a large number of patients were included in our study, CDI was only proven in a subset of patients, thereby potentially affecting statistical analysis. A future prospective multicenter study with a respective sample size calculation addressing these points is thus highly warranted.

Interestingly, CLE allowed the identification of bacteria in the mucosa of patients with CDI *in vivo*. Previously, it was shown by Kiesslich and coworkers that endomicroscopy with topically applied acriflavine could readily identify *Helicobacter pylori* infection *in vivo* [23]. Very recently, the same group demonstrated that endomicroscopy was able to identify intramucosal enteric bacteria *in vivo* in the colon and ileum of patients with ulcerative colitis and Crohn's disease using fluorescein-aided endomicroscopy [19]. It was shown that intramucosal bacteria were more frequently and with a wider distribution found in patients with inflammatory bowel disease than in patients with a normal intestine. However, in our study no bacteria in the colonic mucosa could be identified by fluorescein-aided CLE. Importantly, we were only able to visualize bacteria after application of acriflavine which is a cationic dye that can be detected by its intrinsic fluorescence and accumulates in the endosomal/lysosomal compartment of cells [24]. Thus, our findings suggest that topical administration of acriflavine results in translocation of the dye in the mucosa via epithelial gaps or epithelial erosions followed by its uptake in *C. difficile* bacteria [25]. The fact that these bacteria are negative by fluorescein staining but positive upon acriflavine use is striking and discriminates this bacterial strain from other bacteria previously identified in the colonic mucosa [19]. To our knowledge, this is the first report on a fluorescein-negative bacterial strain in the colonic mucosa by CLE analysis. Thus, your finding of staining patterns is very interesting and may aid in the further classification of different bacteria. Therefore, we suggest that in the future CLE should be evaluated to analyze bacterial cultures of various gram-negative and gram-positive pathogens.

In order to validate the presence of intramucosal *C. difficile* bacteria in patients with CDI, we performed several *ex vivo* studies. Intramucosal bacteria imaged by endomicroscopy *in vivo* looked similar to *C. difficile* in an *ex vivo* cell suspension of pure *C. difficile* culture when analyzed by CLE. Again, only acriflavine-aided endomicroscopy was feasible to detect *C. difficile* by *ex vivo* CLE, while fluorescein-aided endomicroscopy revealed no bacteria. This finding underlines our *in vivo* results where we could only identify bacteria after topical application of acriflavine.

High-magnification of *C. difficile* bacteria, both *in* and *ex vivo* revealed a rod like appearance of these microorganisms. Additionally, there was a strong concordance between the presence of intramucosal bacteria identified *in vivo* and bacteria identified by FISH in biopsies of areas which were previously investigated using endomicroscopy. In order to further highlight the potential of CLE for detection of specific bacteria in CDI, additional *ex vivo* studies on endomicroscopy with previously validated specific molecular probe for *C. difficile* were performed and the results were compared to those of the FISH analysis. By using this approach, we could identify the presence of specific intramucosal *C. difficile* bacteria within the colon in CDI. These findings underline the potential of endomicroscopy for molecular imaging of CDI and specific detection of *C. difficile* bacteria. By using *ex vivo* CLE with the *C. difficile* specific probe we have shown that the probe seems to be able to penetrate the cells without the use of previous fixation. Our hypothesis is that the mucosal structure of the inflamed tissue (according to *C. difficile* colitis) allows penetration of the probe. This speculation is underlined by recent studies evaluating *in vivo* confocal imaging in patients with inflammatory bowel diseases. These studies suggested that patients with colitis has an increased number of epithelial gaps in human small and large intestine and that these gaps must be considered as a component of the intestinal barrier and may therefore have potential implications for intestinal barrier dysfunction in human diseases [25], [26].

Our findings could have a substantial clinical impact, as endomicroscopy has the potential to diagnose *C. difficile* infection *in vivo,* thus potentially enabling an accelerated diagnosis and an improved patient management. Therefore, endomicroscopy might have the potential to refine our understanding of *C. difficile* diagnosis and may help to prevent nosocomial transmission of this life-threatening disease. Moreover, we were able to describe intramucosal bacteria *in vivo*. The clinical significance of our findings should be elucidated in future prospective studies.
